# Evaluating the role of body size and habitat type in movement behavior in human‐dominated systems: A frog's eye view

**DOI:** 10.1002/ece3.9022

**Published:** 2022-06-22

**Authors:** Mason Murphy, Michelle Boone

**Affiliations:** ^1^ Department of Biology Miami University Oxford Ohio USA

**Keywords:** Anura, desiccation, dispersal, habitat edges, land‐use, orientation

## Abstract

1. Animal movement is a key process that connects and maintains populations on the landscape, yet for most species, we do not understand how intrinsic and extrinsic factors interact to influence individual movement behavior.
2. Land‐use/land‐cover changes highlight that connectivity among populations will depend upon an individual's ability to traverse habitats, which may vary as a result of habitat permeability, individual condition, or a combination of these factors.
3. We examined the effects of intrinsic (body size) and extrinsic (habitat type) factors on desiccation tolerance, movement, and orientation in three anuran species (American toads, *Anaxyrus americanus*; northern leopard frogs, *Lithobates pipiens*; and Blanchard's cricket frogs, *Acris blanchardi*) using laboratory and field studies to connect the effects of susceptibility to desiccation, size, and movement behavior in single‐habitat types and at habitat edges.
4. Smaller anurans were more vulnerable to desiccation, particularly for species that metamorphose at relatively small sizes. Habitat type had the strongest effect on movement, while body size had more situational and species‐specific effects on movement. We found that individuals moved the farthest in habitat types that, when given the choice, they oriented away from, suggesting that these habitats are less favorable and could represent barriers to movement.
5. Overall, our work demonstrated that differences in habitat type had strong impacts on individual movement behavior and influenced choices at habitat edges. By integrating intrinsic and extrinsic factors into our study, we provided evidence that population connectivity may be influenced not only by the habitat matrix but also by the condition of the individuals leaving the habitat patch.

## Graphical abstract

We examined the effects of intrinsic (body size) and extrinsic (habitat type) factors on desiccation tolerance, movement, and orientation in three anuran species using a combination of laboratory desiccation trials and field studies to disentangle the individual and combined effects of these factors on movement. Habitat type had the strongest effect on movement, while body size had more situational and species‐specific effects on movement. We found that the habitats that most individuals oriented away from were the ones in which that same species moved the farthest, suggesting that this habitat is less favorable and could represent a barrier for movement.

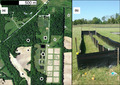

## 1 INTRODUCTION

Nathan et al.’s (2008) movement paradigm highlighted that an organisms' ability or willingness to move is influenced by both intrinsic factors, such as individual body size (Bonte & de la Pena, 2009; Jenkins et al., 2007; Yagi & Green, 2017), and extrinsic factors, such as the landscape matrix (Baguette et al., 2013; Cushman, 2006; Gibbs, 1998). Thus, an individual's movement is affected by the interplay between the internal state and external factors, so that the habitat matrix can promote isolation or connectivity based on an individual's behavioral response or its likelihood to successfully traverse the landscape (Jønsson et al., 2016; Kuefler et al., 2010). Determining how body size and habitat type individually and in combination influence movement behavior in complex environments could illuminate the dynamics of species‐specific movement between populations.

Larger individuals have increased physical advantages and energetic resources (Arribas et al., 2012; Rundle et al., 2007; Yagi & Green, 2017), which allows them to move greater distances and reduces predation and desiccation risks relative to small individuals (Travis et al., 2012). In this way, body size and condition may be a predictor of individuals most likely to successfully disperse and increase population connectivity (Benard & McCauley, 2008; Nathan et al., 2008). Further, size influences desiccation tolerance in some species, which has been shown to be a primary driver of species movement and distributions, with desiccation‐tolerant species more likely to successfully move between habitats (Havel et al., 2014; Mänd et al., 2007; Watling & Braga, 2015; Werner, 1986).

The habitat type can also influence individual movement decisions (Zollner & Lima, 2005). For instance, some forest‐associated species orient away from agricultural fields, clear‐cut forests, or roads, which may be attributed to less vegetation structure, lack of available cover, and/or increased desiccation risk (Cline & Hunter, 2014; Martin et al., 2020; Rothermel & Semlitsch 2002; Schwarzkopf & Alford, 1996). These avoidance behaviors can strongly affect the movement of individuals, limiting movement for resource acquisition, predator avoidance, and population connectivity (Espinosa et al., 2018; Olah et al., 2017; Peterman et al., 2014). While many instances of land‐use change can generate barriers and create a habitat matrix that restricts movement through unfavorable habitats (Gibbs, 1998; Kuefler et al., 2010), habitat change can also connect other populations (Öckinger et al., 2012; Youngquist & Boone, 2014). While research examining movement behavior has advanced our knowledge of population connectivity at a landscape scale (Baguette et al., 2013; John‐Alder & Morin, 1990), few studies have attempted to decouple the roles of body size and habitat type on dispersal and movement (though see Hawkes, 2009).

Pond‐breeding amphibians are an ideal study system to explore the effects and interactions of individual condition and habitat type on movement behavior. Many pond‐breeding amphibians exist in metapopulations (Smith & Green, 2005), which are characterized by spatially distinct subpopulations connected by some level of recurring, yet limited, asynchronous dispersal and gene flow (Hanski, 1998; Marsh & Trenham, 2001). Anuran movement capability has been shown to be sensitive to body size (Cayuela et al., 2020) with larger individuals exhibiting increased jumping distance and endurance in both adult and juvenile anurans (Boes & Benard, 2013; Cabrera‐Guzmán et al., 2013; Yagi & Green, 2017). Composition and configuration of habitat can alter movement patterns and orientation behavior at habitat edges (Mazerolle, 2001; Younquist & Boone, 2014), influencing overall population connectivity. Additionally, amphibian movements have also been tied to local weather patterns, with increased movement during warmer, wetter periods (Todd & Winne, 2006). Furthermore, previous studies have examined amphibian movement responses to habitat type and found differences in both habitat preference and movement length between species (Denoël et al., 2018; Mazerolle, 2001; Rothermel & Semlitsch, 2002), and overall differences in habitat permeability (Arntzen et al., 2017; Van Buskirk, 2012).

We conducted a set of experiments to evaluate the links between body size, desiccation tolerance, movement behavior in single habitats, and movement and initial orientation at habitat edges in juveniles of three species of anurans: American toads (*Anaxyrus americanus*), northern leopard frogs (*Lithobates pipiens*), and Blanchard's cricket frogs (*Acris blanchardi*). These three anurans were selected because they differ in size at metamorphosis from small to large (American toads < cricket frogs < northern leopard frogs) and vary from open‐canopy associated (northern leopard frogs and cricket frogs) to more forest‐associated (American toads). Though the specific movement responses may differ between adult and juvenile anurans in both habitat specificity (Jenkins et al., 2006) and due to size differences (Todd & Winne, 2006), juveniles are thought to be the primary dispersal stage for many pond‐breeding amphibians (Pittman et al., 2014; Semlitsch, 2008; though see Smith & Green, 2006), and thus a critical stage at which to assess the impacts of both intrinsic and extrinsic factors.

Our study addressed the central question: how do habitat and body size, individually and in tandem, affect the movement and orientation of anurans? We hypothesized that movement behavior through habitat types will be influenced by susceptibility to desiccation, which is influenced by cover type and body size within and across species. As our species vary in preferred habitat type, we predicted species‐specific movement patterns between single‐habitat types, and species‐specific orientation toward preferred habitat (e.g., forest for American toads, and old field for northern leopard frogs and cricket frogs). Given a smaller surface area to volume ratio and increased movement ability, we predicted that a larger body size would increase desiccation tolerance, which would promote longer movement distances in both single‐habitat enclosures and at habitat edges in an open choice setting. Lastly, we predicted that body size and habitat type would interact to create size‐specific movement patterns in single habitats and would reduce the strength of habitat choice at habitat edges.

## 2 METHODS

### 2.1 Animal collection and care

We collected American toad and northern leopard frog eggs from at least eight separate egg strings or masses from ponds near Oxford, OH. We collected partial northern leopard frog egg masses on 21–22 March 2017 and 25 March 2018, and American toad egg strings on 6 April 2017 and 8 April 2018. To obtain Blanchard's cricket frog eggs, we collected a total of eight amplexed pairs of cricket frogs near Oxford, OH on 15 and 17 May 2017, which were held overnight in plastic containers with 3 cm of water and twigs for egg deposition. The next day, we collected eggs from each container, and mixed eggs from all pairs to incorporate genetic variation (Semlitsch & Boone 2009).

Eggs from all species were hatched in a temperature‐controlled environment (23°C), and larvae were subsequently transferred to artificial pond mesocosms per species at Miami University's Ecological Research Center (ERC) roughly a week after egg collection (30 March 2017 and 2 April 2018 for northern leopard frogs, 13 April 2017 and 17 April 2018 for American toads, and 23 May 2017 for Blanchard's cricket frogs) and held until metamorphosis. Each mesocosm was set up 2–4 weeks prior to use and contained 1000 L water, 1 kg mixed leaf litter, zooplankton/algae inoculate and were covered with 2 mm mesh lids to prevent the introduction of other species (Hoskins et al., 2019; Semlitsch & Boone, 2009).

For all experiments, we raised tadpoles at density treatments of 20 and 60 individuals per mesocosm to create two distinct size classes of juveniles, large and small, respectively. These densities are within the normal range of densities found in nature and have been used previously to generate distinct size classes (Boone & James, 2003; Pintar & Resetarits, 2017; Semlitsch & Caldwell, 1982). Once tadpoles metamorphosed at Gosner stage 42 (Gosner, 1960); beginning on 19 May 2017 and 21 May 2018 for American toads, 6 June 2017 and 10 June 2018 for northern leopard frogs, and 26 July for Blanchard's cricket frogs, we removed individuals from mesocosms and allowed individuals to reabsorb their tails (Gosner stage 46) in the laboratory. We weighed individuals at Gosner stage 46 and held them in 28 cm × 12 cm × 15 cm terraria containing soil and a water dish at 25°C until enough individuals could be used for desiccation or movement trials. Individuals were held at densities of 10 individuals per terraria and were fed nutrient enhanced (Repticalcium™) crickets ad libitum.

### 2.2 Desiccation tolerance experiment

To assess the intrinsic factor of desiccation tolerance, in2017, we selected 39 individuals of each species ~1 week post‐metamorphosis for northern leopard frogs and cricket frogs, and 3 weeks post metamorphosis for toads, including 15 of each species from the large size class, and 24 from the small size class. We used unequal numbers because we had fewer individuals from larger size classes. Prior to the start of the trials, we gently pressed on the abdomen of each frog to release any fluid in the bladder and then weighed each individual (bench scale, Sartorius AG, resolution 0.001 g). Afterwards, we randomly assigned individuals to either the experimental (10–16 individuals/size class) or control (~5–8 individuals/size class) treatments and placed individuals in 15 × 15 × 10 cm plastic containers with perforated lids on a shelf at 27°C with ambient lighting. We lined control containers with damp paper towels and experimental treatments contained no towel. Following protocols to limit mortality (Rohr & Palmer, 2005; Watling & Braga, 2015), the trials lasted 4 h, and individuals were reweighed to determine mass.

### 2.3 Single‐habitat enclosure experiment

**FIGURE 1:**
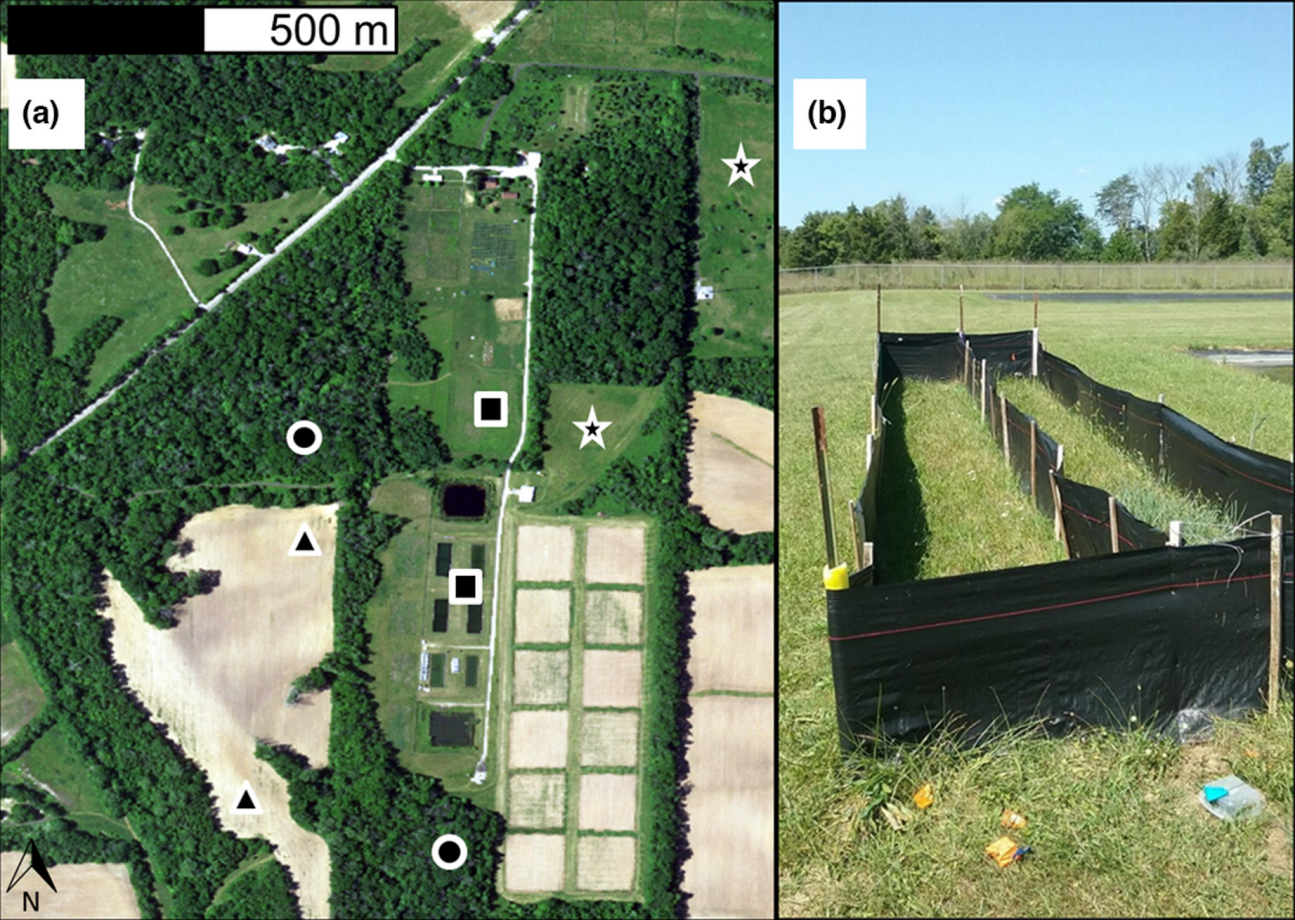
(a) Locations of enclosures at the Ecology Research Center (ERC) in distinct habitat types used in our single‐habitat enclosure experiment (triangle = agriculture, square = mown grass, circle = forest, star = old field). (b) Example of 1 m × 9 m silt fence enclosures in the mown grass habitat at the ERCat Miami University

To test the intrinsic factor of body size and the extrinsic factor of individual habitat type on juvenile anuran movement, we constructed eight 2 m × 9 m × 0.6 m silt fence enclosures in 2017, which were each subdivided lengthwise to create two 1 m × 9 m runs in four distinct habitat types of increasing ground cover (corn agriculture, mown grass, forest, and old field [formerly cultivated land but now dominated by grasses and forbs]) at the ERC (Figure 1). While we did not collect quantitative measures of habitat cover differences, our corn agriculture sites had the least ground cover throughout the experiment, due to prior standard herbicide treatment in previous years. Mown grass was kept cut short (~5 cm) throughout the entirety of the trials. Our forested sites were relatively immature and contained significant ground cover growth and our old field sites contained extremely dense growth of grasses and forbs. The differences between these habitat types are similar to the same general patterns as in other studies of amphibian movement (Cline & Hunter, 2016; Cosentino et al., 2011). We had two replicate enclosures for each habitat type that were oriented perpendicular to each other in separate locations to reduce the probability that individuals were orienting toward a landscape feature. To understand how environmental factors may affect movement, we monitored temperature at each study site hourly using iButton data loggers (Thermochron) placed within 3 meters of each enclosure the day before the first night of tracking. Following the last night of tracking for each species, the iButtons were retrieved and their data downloaded. However, due to damage to the loggers during northern leopard frog and Blanchard's cricket frog trials, temperature data are only included in the American toad dataset.

Each tracking night, we randomly selected 16 individuals of the same species, eight from each size class, and weighed those individuals before tracking. We coated juvenile anurans in fluorescent powder (DayGlo Color Co.) without coating the eyes and mouth (Rittenhouse et al., 2006). We released one individual of each size class on one side of each enclosure run to generate replicates of size class and habitat type. We randomly assigned release corners and allowed individuals 1 min of acclimation underneath an opaque plastic container before release. All individuals were released within 30 min of sunset. We returned to track juvenile movement using handheld black lights 4 h after release. We marked paths by placing numbered flags at each turn of greater than 10° and returned the following day to measure the distance between the flags. We used these measurements to calculate movement metrics including total path distance and displacement (net distance traveled, potentially an indicator of linear movement).

We tracked American toads from 10–19 June 2017, northern leopard frogs from 20–29 June 2017, and Blanchard's cricket frogs from 3–22 August 2017, for a total of 128 individuals per species on 8 nights per species. We analyzed data from a total of 109 American toads, 118 northern leopard frogs, and 95 Blanchard's cricket frogs, after removing individuals from nights with unexpected rain that limited our tracking ability (8 American toads, 8 northern leopard frogs, 16 Blanchard's cricket frogs) and individuals that did not move within the enclosure (Youngquist & Boone, 2014), based on a minimum of 0.5 m of total path movement (11 American toads [8 small, 3 large], 2 northern leopard frogs [2 small, 0 large], 17 Blanchard's cricket frogs [9 small, 8 large]).

### 2.4 Edge‐Choice experiment

**FIGURE 2:**
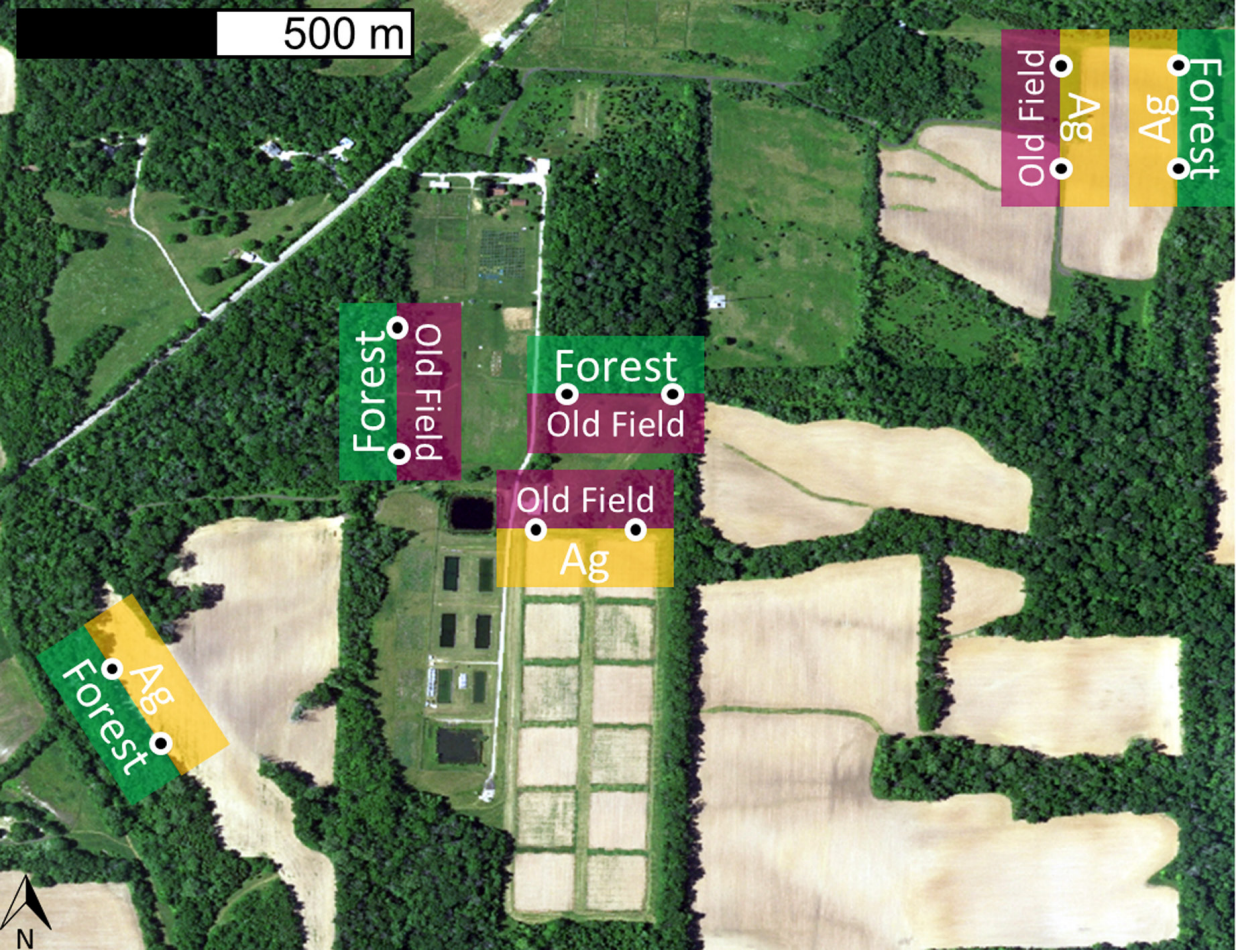
Location of release points (circles) at habitat edges at the Ecology Research Center for our edge‐choice experiment. While the same edge was used for two release points, the distance between release points was greater than the movement distance of released individuals

To test for the impact of body size and habitat type on initial orientation and preference at habitat edges in 2018, we used release sites at the ERC of two combinations of each combination for the three habitat types (forest, old field, and corn agriculture). At each release site, we had two release points—one for a small and one for a large anuran—resulting in a total of 12 release sites with two replicates of each habitat combination (forest/old field; forest/corn; old field/corn, Figure 2). We released one fluorescent powder coated individual, which was randomly assigned to a release point, at each release site within 30 min of sunset over 10 nights per species, for a total of at least 120 released individuals per species, with 20 replicates for each size class and edge type combination. We returned 4 h post‐release to mark movement paths with numbered flags; 4‐h time frames were used to increase the probability that we could recover the individuals and because fluorescent powder trails could not be followed much longer than this time frame. Individuals were recaptured, and orientation direction was assessed from the release point. The initial choice was assigned to a particular habitat if an individual's final location was at least 0.5 m into one of the two habitat types; otherwise, the habitat choice was scored as “edge.” Additionally, we measured the same movement path characteristics as above in the single‐habitat movement study, including total path length, displacement, and orientation angle relative to the edge. We tracked a total of 120 American toads from 24 May to 7 June 2018 and 132 northern leopard frogs from 14 June to July 2018. We did not test Blanchard's cricket frogs because of time constraints and because previous research by Youngquist and Boone (2014) indicated that at the same test locations, Blanchard's cricket frogs avoided forest; however, that study did not include different size classes. Due to unexpected rain, we removed 12 difficult‐to‐track northern leopard frogs and added one extra night of tracking. After removing individuals that did not move or were unable to track, we analyzed the movement and choice of 92 American toads and 118 northern leopard frogs.

### 2.5 Statistical analyses

We examined the effect of larval density treatment on mass at metamorphosis using analysis of variance (ANOVA) in each year of the study separately; the mesocosm was used as the experimental unit. Prior to use in each experiment, we tested the effect of larval density on juvenile mass after short‐term terrestrial rearing using ANOVAs; individuals were used as the experimental unit. In the 2017 desiccation tolerance trials, we examined the differences in both absolute mass and percent change of total body mass before and after desiccation trials using ANOVAs. For the 2017 experiment examining movement in single‐habitat enclosures, we evaluated the effect of habitat type and juvenile size class on three movement variables: total path distance, displacement distance, and path linearity using ANOVAs, with date included as a time block. Temperature, used as an average of hourly recorded temperatures at each site, was also included in the analysis of American toad movement. In the 2018 edge‐choice experiment, we examined the effect of size class on the three‐movement variables, habitat choice, and orientation of both species, with a day of year included as a time block. All data were normally distributed with the exception of total path distance, which was normalized using a log transformation in both edge‐choice and single‐habitat studies. When habitat type was significant, we assessed the estimated marginal means with a Tukey adjustment using the R package emmeans to determine treatment‐level differences. All analyses were conducted in R version 3.5.1 (R Core Team, 2020).

To assess orientation and initial habitat choice in the edge‐choice experiment, we standardized the habitat edges at release points along the 0–180 degree line and then used nonparametric circular statistics to create a mean orientation, which allowed for the assessment of habitat choice and/or preference for the edge. We tested for differences between sites using Watson's two‐sample tests of homogeneity (Watson, 1961), aggregated replicates, and tested for circular uniformity using Watson's one‐sample test for circular uniform distribution (Watson & Williams, 1956). We examined habitat choice with binomial exact tests, analyzing each edge type separately, as all three options were not available for each replicate. We compared differences in habitat choice between size classes using Watson's *U* tests, and also aggregated size classes to assess overall species‐level choice.

## 3 RESULTS

**FIGURE 3:**
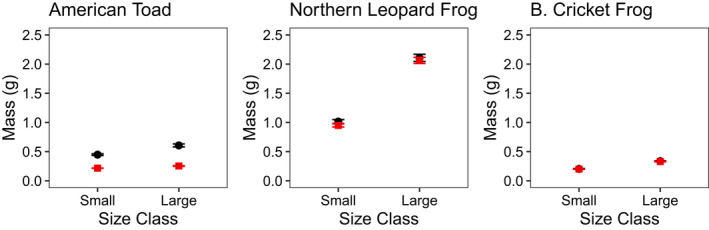
Comparison of mean mass and ± 1 SE between different density treatments at metamorphosis and immediately before tracking in 2017 (single‐habitat enclosure experiment)

Larval density treatments in 2017 resulted in marginally to significantly different size classes for all three species emerging from mesocosms (American toad *F*_1,6_ = 33.3, *p* = .071; northern leopard frog *F*_1,6_ = 168.8, *p* < .001; Blanchard's cricket frog *F*_1,6_ = 128.2, *p* = .008; Figure 3), which persisted to the day of desiccation trials (all *p* < .001, American toad *F*_1,37_ = 23.6, northern leopard frog *F*_1,37_ = 331.0, Blanchard's cricket frog *F*_1,37_ = 71.1) and to the night of tracking (all *p* < .001, American toad *F*_1,107_ = 31.1, northern leopard frog *F*_1,116_ = 258.3, Blanchard's cricket frog *F*_1,93_ = 208.9; Figure 3). Larval density treatments in 2018 also resulted in distinct size classes that persisted to night of tracking (*p* < .001, American toad *F*_1,90_ = 26.7; northern leopard frog *F*_1,116_ = 502.6).

### 3.1 Desiccation trials

**FIGURE 4:**
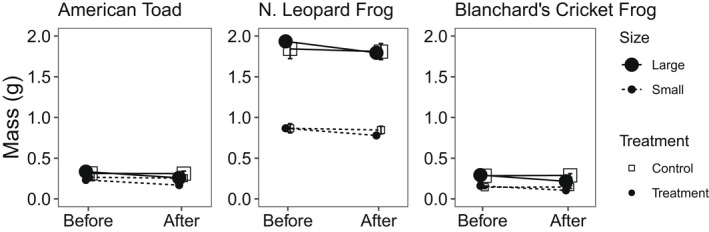
Mean mass ± 1 SE of each species for both treatment and control before and after desiccation experiment

Our desiccation trials resulted in all individuals in the desiccation treatment losing a significant amount of mass (all *p* < .001, American toads *F*_1,35_ = 216.6; northern leopard frogs *F*_1,35_ = 28.4; Blanchard's cricket frogs *F*_1,35_ = 304.8 [Figure 4]) relative to control animals. Furthermore, percent of total body mass lost was significantly greater for small size class individuals than for large size class individuals for American toads (28% vs. 23%, *F*_1,24_ = 4.7, *p* = .040) and Blanchard's cricket frogs (34% vs. 26%, *F*_1,24_ = 17.0, *p* < .001) but not for northern leopard frogs (9% vs. 7%, *F*_1,24_ = 3.1, *p* = .093; Figure 4).

### 3.2 Single‐Habitat enclosure experiment

**FIGURE 5:**
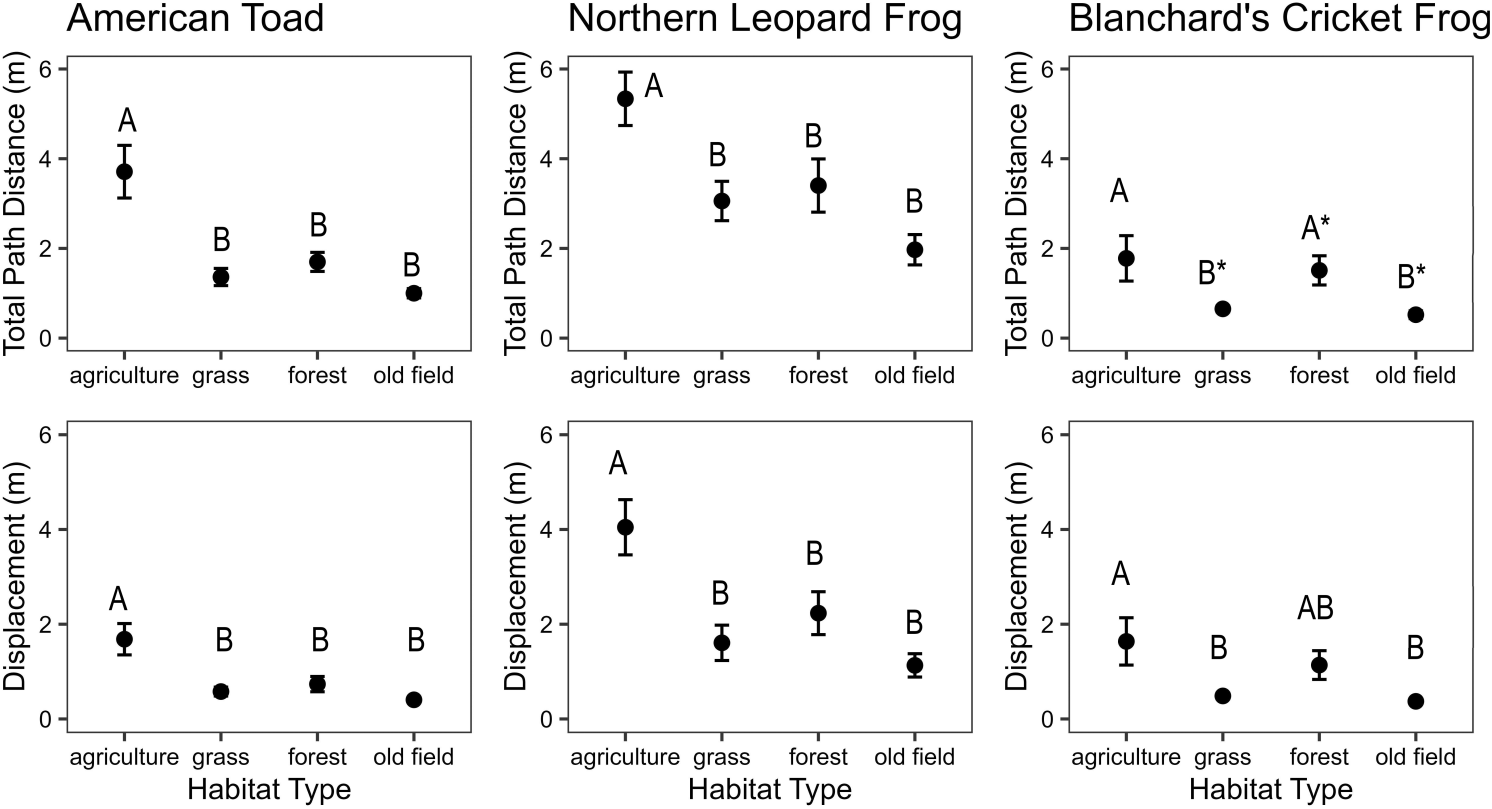
Mean ± 1 SE total path distance and displacement distance for each species in all four habitat types (all *p* < .05). Letters represent groups based on the Tukey's post hoc test (*p* < .05); *represents marginally significant differences (*p* < .1, single‐habitat enclosure experiment)

The extrinsic factor habitat type explained the greatest amount of variation in the movement for all three species. Habitat type significantly affected total path distance and displacement distance in all species, except for displacement in Blanchard's cricket frogs where it had a marginally significant effect (Table 1 and Figure 5).

**TABLE 1:** Univariate statistical output of ANOVA tests for the effect of habitat, size class, and their interaction on three movement responses in our single‐habitat enclosure experiment

Species	Response	Source of variation	*df*	*F*	*p*
American toad	Total path distance	**Habitat**	**3**	**14.121**	**<.001**
		Size class	1	1.051	.308
		Temperature	1	0.005	.943
		Date	1	1.015	.316
		*Habitat X Size class*	*3*	*2.519*	*.062*
		Error	99		
	Displacement	**Habitat**	**3**	**10.816**	**<.001**
		**Size Class**	**1**	**5.963**	**.016**
		Temperature	1	0.452	.502
		Date	1	1.765	.187
		**Habitat X Size class**	**3**	**2.861**	**.041**
		Error	99		
Northern leopard frog	Total path distance	**Habitat**	**3**	**5.7533**	**<.001**
		Size class	1	0.0098	.921
		**Date**	**1**	**8.4829**	**.004**
		Habitat X Size class	3	0.665	.575
		Error	109		
	Displacement	**Habitat**	**3**	**6.225**	**<.001**
		Size class	1	0.008	.931
		**Date**	**1**	**7.014**	**.009**
		Habitat X Size class	3	0.236	.871
		Error	109		
Blanchard's cricket frog	Total path distance	**Habitat**	**3**	**3.175**	**.028**
		Size class	1	0.074	.786
		Date	1	0.897	.346
		Habitat X Size class	3	0.539	.656
		Error	86		
	Displacement	*Habitat*	*3*	*2.293*	*.083*
		Size class	1	0.034	.853
		Date	1	0.249	.618
		Habitat X Size class	3	0.248	.862
		Error	86		

*Note*: Results in bold are significant (*p* < .05), and results in italic are marginally significant (.05 < *p* < .1).

Northern leopard frogs and American toads moved significantly longer total path distances in agricultural habitats compared with all other habitat types. Similarly, Blanchard's cricket frogs moved significantly farther in agriculture and forest than they did in grass or old field habitats (Figure 5). Total displacement generally followed the same pattern as total path distance for all species across habitats, indicating that when species traveled longer distances it typically resulted in a greater displacement distance—the distance from their starting and stopping points (Figure 5).

Average temperature and standard errors during American toad trials were 21.89 ± 0.28°C for agriculture, 21.54 ± 0.24°C for forest, 21.06 ± 0.39°C for mown grass, and 19.91 ± 0.32°C for old field. Though temperature did significantly differ between sites (*p* < 0.001), this was driven largely by the lower average temperatures found in the old field habitat (Tukey's HSD *p* < 0.05 for habitat temperature comparisons with old field and *p* > 0.05 between other habitat types). Furthermore, the temperature did not significantly affect either movement measure in American toads. Tracking day, our time block, had no effect on either of the movement metrics in either American toads or Blanchard's cricket frogs; however, our time block did significantly affect northern leopard frog total path distance and displacement (Table 1).

Size class affected the movement of American toads but not northern Leopard frogs or Blanchard's cricket frogs (Table 1). Specifically, size class influenced American toad displacement, with the smaller size class having a 130% longer mean displacement, despite larger size class toads having slightly longer mean total path distances (Small: 1.91 m ± 0.272, large: 2.04 m ± 0.280), resulting in more directed and linear movement for smaller size class toads.

**FIGURE 6:**
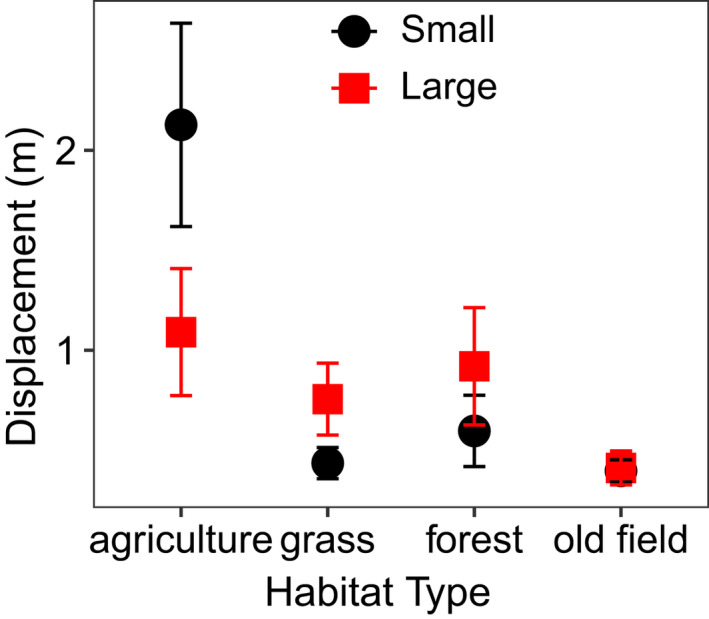
The impact of habitat type across size classes on mean ± 1 SE displacement in American toads (*p* = .043, single‐habitat enclosure experiment)

We found a significant interaction between habitat type and size that affected American toads' displacement distance (Table 1). Smaller toads exhibited greater overall mean displacement distance relative to larger individuals, but this was driven exclusively by very high displacement in corn agriculture habitat; larger toads showed similar or greater displacement distances in all other habitat types (mean displacement in forest; 0.92 ± 0.293 m vs. 0.597 ± 0.178 nm, grass; 0.756 ± 0.179 m vs. 0.436 ± 0.077 m; and old field 0.412 ± 0.084 vs. 0.397 ± 0.055 m; Figure 6).

### 3.3 Edge‐Choice experiment

In the edge‐choice experiment, the intrinsic factor size class was an important predictor of movement variables for northern leopard frogs and American toads (Table 2). However, size class did not affect orientation and initial choice in any habitat combination for either species (Table 3). Larger size class northern leopard frogs had longer total path distances (141%), and marginally greater displacements (Table 2). Although the size class effect on displacement in American toads was marginal (Table 2), it followed the same pattern as our previous experiment, with smaller toads exhibiting greater displacement.

**TABLE 2:** Univariate statistical output of ANOVA tests for the effects of size class on the movement for each species in edge‐choice experiment with date of the trial used as a block

Species	Response	*df*	*F*	*p*
American toad	Total path distance	1	1.17	.282
	Date	1	1.48	.231
	*Displacement*	*1*	*3.81*	*.054*
	Date	1	0.38	.538
	Error	89		
Northern leopard frog	**Total path distance**	**1**	**13.54**	**<.001**
	**Date**	**1**	**12.58**	**<.001**
	*Displacement*	*1*	*3.10*	*.081*
	**Date**	**1**	**6.96**	**.010**
	Error	115		

*Note*: Results in bold are significant (*p* < .05), results in italic are marginally significant (.05 < *p* < .1).

**TABLE 3:** Watson's test of size differences in mean orientation at habitat edges

Species	Edge	Watson's *U* ^2^	*p*
American toad	Forest/agriculture	0.1207	>.10
American toad	Forest/old field	0.0426	>.10
American toad	Old field/agriculture	0.0613	>.10
Northern leopard frog	Forest/agriculture	0.0545	>.10
Northern leopard frog	Forest/old field	0.144	>.10
Northern leopard frog	Old field/agriculture	0.1353	>.10

**FIGURE 7:**
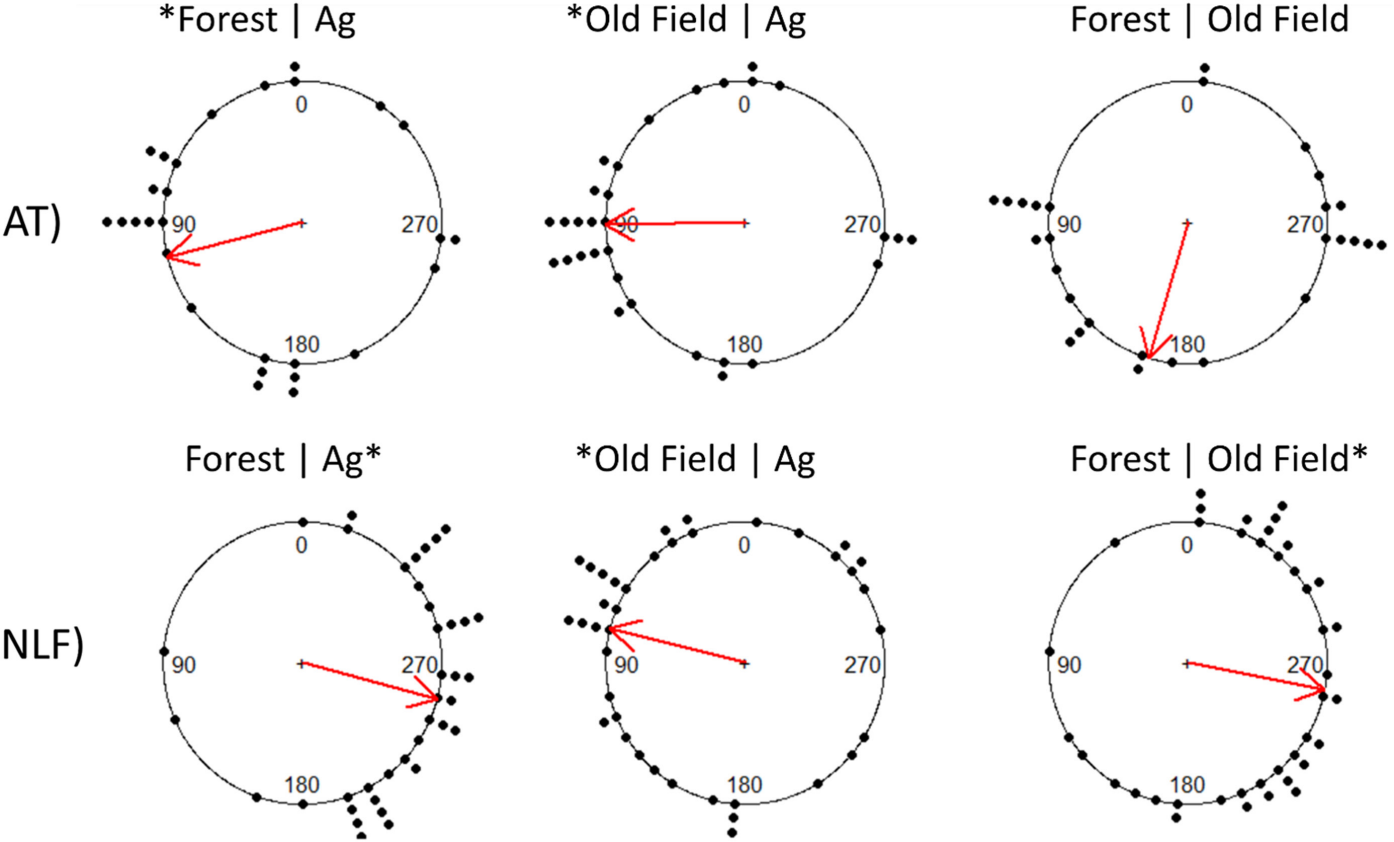
Mean orientation traveled by American toads (AT) and northern leopard frogs (NLF) in edge‐choice experiment. Dots around the circle represent the angle of travel for one or more individual frogs, and arrows represent mean angle of travel. Left and right halves of the circle represent different habitat types. * indicates significant (*p* > .05) orientation direction different from the uniform distribution (via Watson's uniformity tests), and the mean angle of significant unimodal clustering is within the habitat type (via binomial tests)

When size classes were aggregated, we found nonuniform circular distributions for toads at the forest/agriculture edge (*U* ^2^ = 0.1957, *p* < .05), and the old field/agriculture edge (*U* ^2^ = 0.4216, *p* < .01) but not the forest/old field edge (*U* ^2^ = 0.1414, *p* > .10). We found nonuniform circular distributions for leopard frogs at all edge types ([forest/agriculture, *U* ^2^ = 0.7508, *p* < .01], [old field/agriculture, *U* ^2^ = 0.2370, *p* < .025], [forest/old field, *U* ^2^ = 0.3086, *p* < .01]). Each species exhibited species‐specific differences in habitat choice with American toads avoiding corn agriculture habitat and northern leopard frogs avoiding forest habitat (Figure 7). Overall initial habitat preference for American toads was forest = old field > agriculture, and for northern leopard frogs it was old field > agriculture > forest.

## 4 DISCUSSION

Our study provides evidence that the relative importance of the intrinsic factor body size differed among three species of pond‐breeding anurans and could impact movement ability and desiccation tolerance in smaller‐bodied species. However, the extrinsic factor habitat type overall had a stronger impact on individual movements. Combined, these results suggest that choices individuals make at habitat edges and their ability to move through less preferred habitat quickly not only results in species‐specific patterns of population connectivity across the same landscape but may also allow some populations to be more connected in matrixes with less favorable intervening terrain than we would predict based solely on species' preferences (Arens et al., 2007; Langone et al., 2016; Sinsch, 2014).

### 4.1 Habitat type affects movement

Short movement distances may indicate an inability to navigate and cross that habitat, or conversely show a willingness to remain in and seek out resources in a favorable habitat (Fahrig, 2007; Hawke et al., 2021; Leblond et al., 2010). For instance, when in preferred habitat, individuals display resource and shelter‐seeking movements, which are slower and less linear in nature than escape or dispersal movements (Bowler & Benton, 2005; Semlitsch, 2008). Likewise, long‐distance movement across a landscape could be indicative of a strong ability to navigate and cross a habitat, or an attempt to exit unfavorable habitat (Buderman et al., 2016; Fahrig, 2007; Semlitsch et al., 2008). For example, some species, such as red squirrels (*Sciurus vulgaris*) and wood frogs (*Lithobates sylvaticus*) have shown limited, the rapid crossing of nonpreferred habitats (Bakker & Van Vuren, 2004; Cline & Hunter, 2016). Gap crossing, which is typified by rapid directional movement (Bowman & Fahrig, 2002), can be limited by individual and environmental constraints (Bakker & Van Vuren, 2004; Bright, 1998; Hillaert et al., 2020). Similar rapid linear movement is also seen in evacuation behavior, in which individuals demonstrate rapid movement behavior when placed in unfavorable habitat in attempts to move to more favorable habitats, such as forest‐associated species including American toads and ringed salamanders (*Ambystoma annulatum*) rapidly evacuating habitat following clear‐cuts (Escobar & Estades, 2021; Semlitsch et al., 2008).

The extrinsic factor habitat type strongly impacted movement in all species, and by examining movement in both single habitats and at edges, we were able to assess a more complex relationship of habitat with movement beyond a single movement parameter. We showed that low levels of movement in single‐habitats corresponded to initially preferred habitat in edge‐choice experiments, and that high levels of movement were exhibited in nonpreferred habitats in juveniles of both American toads and northern leopard frogs. Youngquist and Boone (2014) examined the orientation behavior of juvenile Blanchard's cricket frogs using the same habitat edge types as our study and found that they, like northern leopard frogs, avoided forested habitats, although they exhibited no strong choice behavior between agriculture and old field habitats.

Coupled with the results of our edge‐choice experiment, which showed that agriculture was avoided by American toads and a second choice for northern leopard frogs, our data suggest that rapid, lengthier movements are a result of individuals attempting to leave or move quickly through the unfavorable habitat. Suitable habitat may not always correspond to the perceived permeability of specific habitat (Kuefler et al., 2010; Morris et al., 2004). Indeed, while we did not test mown grass habitat in our edge‐choice experiment, old field habitat was consistently the top initial movement choice by both northern leopard frogs and American toads over agriculture habitat. We found low overall movement in preferred habitat, highlighting that while individuals may have the capacity to move across unfavorable habitat rapidly, movement may be largely or preferably restricted to slower, more tortuous movement through preferred habitat (McClure et al., 2016; Schtickzelle & Baguette, 2003; Valenzuela‐Sánchez et al., 2019).

Strong orientation behaviors at habitat edges suggest that even with high movement ability, forested habitats may serve as barriers for dispersal in open‐canopy species such as northern leopard frogs (present study) and Blanchard's cricket frogs (Youngquist & Boone, 2014), just as agricultural habitat may serve as barriers for American toads (present study). Habitat edges are recognizable by a wide variety of organisms, eliciting movement behavior responses (Cline & Hunter, 2014; Stevens et al., 2006). Changes in movement behavior and orientation due to the extrinsic factor habitat edges can result in edges forming effective barriers for dispersal, limiting connectivity even if suitable habitat patches might be close in Euclidean distance (Cayuela et al., 2020; DeMaynadier & Hunter, 1999). In effect, habitat edges serve as barriers for movement, though unfavorable habitats can be moved through rapidly in an attempt to avoid remaining in that unfavorable habitat.

### 4.2 Body size affects desiccation risk

Larger individuals often exhibit increased movement distance and endurance (Cabrera‐Guzmán et al., 2013; Eckert et al., 2008; Hyslop et al., 2014), and generally have a lower risk of desiccation (Hillman et al., 2000; Tracy et al., 2010) due to a lower body surface to volume ratio. In our study, however, the size of juveniles shortly after metamorphosis did not affect movement or habitat choice as profoundly as expected across all species, particularly given that both small American toads and small Blanchard's cricket frogs lost a greater proportion of their body weight in the desiccation study relative to larger juveniles.

American toads and Blanchard's cricket frogs are relatively small at metamorphosis and smaller individuals would be more vulnerable in dry environments, a potentially interesting life history tradeoff (Einum et al., 2012; Russell et al., 2005). Differences in desiccation between size classes suggest that individuals may have size‐specific movement behaviors and make different choices between habitats based on desiccation risk. Indeed, size class affected some parameters of American toad movement in interaction with habitat type in our single‐habitat enclosures. Even so, we observed no substantial effect of size on orientation in our edge‐choice experiment when nonpreferred habitat could be behaviorally avoided. Northern leopard frogs reached the largest size at metamorphosis of species in this study, yet neither the small or large groups lost much relative mass during the desiccation trial, nor did body size have any effects on overall movement. Nevertheless, the relationship between movement and body size may not be linear. Yagi and Green (2017) found for Fowler's toads (*Anaxyrus fowleri*), the largest movements were made by individuals of intermediate size. Given this relationship, our study may have been unable to discern the full relationship using only two size classes.

### 4.3 The interaction of habitat and body size can generate complex patterns of movement

Both northern leopard frog juveniles (present study) and Blanchard's cricket frog juveniles (Youngquist & Boone, 2014) made an initial movement into agricultural habitat over forested habitat and showed the highest levels of movement in agriculture. While this suggests cropland is a highly permeable habitat type for amphibian movement, given the relative distances traveled compared with other habitats, the actual usage of this habitat for movement may impose size‐specific and species‐specific penalties (Jacob et al., 2020). Agriculture habitat can pose a desiccation risk for amphibians (Vos et al., 2007), especially in corn agriculture (Cosentino et al., 2011), and the interaction of intrinsic and extrinsic factors suggests that size‐specific desiccation risk could limit the ability of Blanchard's cricket frogs to cross agriculture habitat. Graeter et al. (2008) examined the movements of three species in two habitats and found that northern leopard frogs oriented and used clear‐cut habitat when soil moisture was high, suggesting that with larger movements, larger individuals may be able to have more success in gap‐crossing movements relative to other species. Temperature can affect movement ability in other ectotherms (Mitchell & Bergman, 2016), and is therefore important to account for when assessing any measure of amphibian movement. While we did find temperature differences between habitat types while tracking toads, night temperature during tracking did not significantly affect any measure of movement.

Though we found only modest effects of body size on movement in our studies, we did observe an interaction between body size and habitat type in American toads. Small toads, which had the greatest desiccation risk, showed the greatest displacement in corn agriculture habitats—the habitat where they would be most vulnerable relative to larger toads, yet in all other habitats larger toads had greater displacement as we would expect (Cayuela et al., 2020). Further, in both field experiments, smaller toads exhibited high displacement distances relative to total path distances, suggesting that smaller individuals, more so than larger individuals, were using straight movement paths to more efficiently escape less desirable habitats (Peterman et al., 2011; Rothermel & Semlitsch, 2002; Semlitsch et al., 2008).

While in American toads the keratinized skin typical of adult toads takes longer than three weeks to develop, and keratinization may increase American toad desiccation tolerance (Pfingsten et al., 2013). This study, however, focused on initial movement and may therefore not capture the complete range of accessible movement/dispersal options utilized by more developed toads later in the season. Overall, species with small size at metamorphosis may be particularly susceptible to desiccation and therefore may make these species more sensitive to environmental conditions post‐metamorphosis and more likely to respond to extrinsic factors that may relate to or prevent water loss, especially considering the lack of distinct orientation behavior at habitat edges between size classes (Álvarez & Nicieza, 2002; Pough & Kamel, 1984). Broadly speaking, even though species may share a set of traits that influence their movement behavior, such as desiccation susceptibility or predator avoidance (Cayuela et al., 2020), species‐specific differences in life history and behavior can affect responses to habitat edges (Graeter et al., 2008; Jacob et al., 2020), resulting in different patterns of connectivity across the same landscape.

## 5 CONCLUSIONS

Understanding individual movements can be useful in deciphering both fine and broad‐scale patterns, but it is challenging to study fine‐scale movements of a sufficient number of individuals to directly observe the cumulative effects of individual movement on overall population connectivity. By exploring desiccation, individual‐level movement patterns in different habitats, and orientation at habitat edges, we demonstrated that habitat type represented a critical extrinsic factor that affected multiple facets of movement and orientation in three pond‐breeding species with distinct habitat preferences. Furthermore, we showed that body size influenced intrinsic desiccation risk and some aspects of movement in tandem with habitat type, including increased movement by small individuals in nonpreferred habitats. Yet, overall habitat type explained more variation in movement behavior than juvenile body size. Differences within and between species in movement and orientation at habitat edges suggest that the composition of land cover not only affects species differently but can affect a single species differently between individuals of different body sizes, highlighting a complex group of factors that should be considered when assessing movement, dispersal, and connectivity. By examining the interplay between these factors, we can better inform models of dispersal that account for individual differences in movement within a population, and ultimately create more accurate predictions of the variance in population connectivity due to these differences. In progressively more fragmented landscapes, especially agricultural landscapes with distinct habitat edges, understanding the response of organisms to land‐use change, distinct habitat types, and habitat edges further our understanding of habitat permeability and functional connectivity and enables deliberate and species‐specific responses to mitigate population declines.

## AUTHOR CONTRIBUTIONS

**Michelle Boone:** Conceptualization (equal); data curation (supporting); formal analysis (supporting); funding acquisition (lead); investigation (supporting); methodology (equal); project administration (lead); resources (lead); software (supporting); supervision (lead); validation (equal); visualization (equal); writing – original draft (supporting); writing – review and editing (supporting). **Mason Murphy:** Conceptualization (equal); data curation (lead); formal analysis (lead); funding acquisition (supporting); investigation (lead); methodology (equal); project administration (equal); resources (supporting); software (lead); supervision (supporting); validation (equal); visualization (equal); writing – original draft (lead); writing – review and editing (lead).

## CONFLICT OF INTEREST

None declared.

## Data Availability

Data are deposited in the Dryad Digital Repository (https://doi.org/10.5061/dryad.wstqjq2m2).
